# Exploring dental professionals’ experiences of interprofessional collaboration with home care services

**DOI:** 10.2340/aos.v85.45934

**Published:** 2026-04-24

**Authors:** Ingrid Volden Klepaker, Lena Fauske, Marte-Mari Uhlen-Strand

**Affiliations:** a Oral Health Centre of Expertise in Eastern Norway, Oslo, Norway; b Department of Oncology, Norwegian Radium Hospital, Oslo University Hospital, Oslo, Norway

**Keywords:** interprofessional collaboration, public dental service, home care service, qualitative research

## Abstract

**Aim:**

To explore dental professionals’ experiences of interprofessional collaboration between the public dental service (PDS) and home care services (HCS) in Norway, with a focus on how structural, legal, and professional factors influence access to preventive oral health care for HCS users.

**Materials and methods:**

A qualitative design was applied, informed by phenomenological and hermeneutical approaches. Eight semi-structured interviews were conducted with dental professionals in PDS in Eastern Norway. Data were analyzed thematically using an inductive approach.

**Results:**

Three main themes were identified: (1) *Systemic and practical barriers*, including fragmented administrative structures, outdated legislation, and lack of shared digital infrastructure; (2) *Challenges in interprofessional collaboration*, such as weak institutional anchoring, unclear role expectations, and low prioritization of oral health within HCS; (3) *Prevention of oral disease*, wherein participants emphasized the need for early intervention, continuity, and integration of oral health into daily care routines. Joint home visits and stable interprofessional relationships were highlighted as promising.

**Conclusion:**

Despite national strategies supporting integrated care, structural fragmentation and role ambiguity hinder effective collaboration. Strengthening interprofessional frameworks, updating legislation, and improving digital systems are needed to enhance access to preventive oral health care services. Oral health must be recognized as a shared responsibility across health services.

## Introduction

By 2050, the global population of people aged 60 and above is expected to double [1]. This demographic shift will increase the strain on healthcare services and demand targeted prevention of oral diseases, and prioritizing resources to maintain good oral health. At the same time, improved oral health trends and declining caries prevalence among older adults in Norway have led to longer retention of natural teeth [2, 3], leaving more teeth at risk of caries in later life. Moreover, global evidence indicates that dental caries remains highly prevalent among older adults, especially in vulnerable populations [4].

Poor oral health is found to be higher among people receiving home care, compared to residents in nursing homes [5, 6]. Despite having a statutory right to access the public dental service (PDS) [7], only 15% of individuals in the patient group ‘elderly, chronically ill, and disabled receiving home nursing care’, were examined or treated in 2024 [8, 9]. This represents a critically low number of patients enrolled in PDS, especially considering risks related to oral diseases [10].

In Norway, home care services (HCS) and PDS are organized within different parts of the public administration [11, 12]. These two specific health services are governed by separate legislation and regulations, although overarching policies such as the Coordination Reform [13] and the Public Health Act [14] emphasize efficient resource utilization for integrated care to people receiving HCS.

Healthcare services bear the responsibility of informing users about their rights, given their comprehensive insight into users’ circumstances and needs. The division of responsibility is formalized through a cooperation agreement between HCS and PDS [15]. Nevertheless, many home care users report not receiving information about the available dental services [16]. Similar views were expressed in interviews with chief dental officers in PDS [17]. Similar findings were supported by Schluter et al. [18], where only 25.3% of older adults receiving home care had a dental examination in the past year, indicating barriers targeting this particular part of the population.

Structural government separation and differences in administrative responsibilities can create barriers to effective collaboration between HCS and PDS [16]. At the same time, health care services at different government levels are expected to collaborate at a high level to ensure integrated health care services to the public [13].

Previous research has shown that improved interprofessional collaboration between home care professionals and dental professionals is essential in the prevention of oral diseases among older adults receiving home care [19]. Prevention of disease is one of the main tasks both for HCS and PDS according to legislation and political documents [11, 12, 20, 21]. Simultaneously, it has been shown that dental professionals are more often required to provide acute oral care due to the significant burden of untreated oral disease within this population [22]. This highlights the need for early preventive measures; however, reaching these users remains challenging, as previous research identifies this as a barrier to collaboration [16].

Luhmann’s social systems theory describes systems as operationally closed yet cognitively open, meaning they respond to environmental stimuli through internal structures rather than external forces [23]. Systems create meaning self-referentially and distinguish themselves from their environment. In collaboration between health services, professionals interpret information according to system-specific logic; thus, collaboration is shaped not only by information content but also by the receiver’s interpretive framework, which may contribute to collaboration challenges [24].

This study aimed to explore dental professionals’ experiences of the collaboration between PDS and HCS, and to explore this collaboration from various perspectives through a qualitative lens.

## Materials and methods

A qualitative design was employed in this study, utilizing semi-structured interviews to address the research question. A phenomenological and hermeneutical research approach was used. Phenomenology allows for a deeper understanding of the phenomenon from the participants’ point of view. Combined with a hermeneutical approach, which seeks to interpret and make sense of the participants’ experiences, this enables an in-depth exploration of the participants [25]. Individual interviews were conducted to elicit detailed and nuanced accounts, allowing participants to share both positive and negative experiences in a secure and supportive setting [26, 27].

### Sampling and recruitment

**Figure 1:**
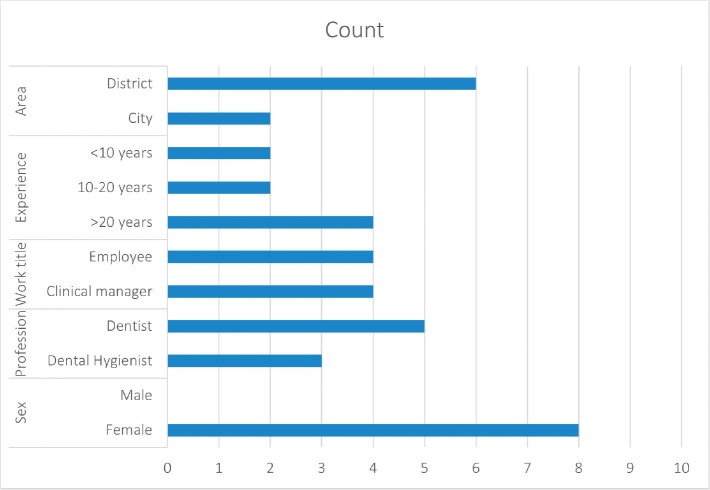
Background characteristics presented in Figure 1 (n=8).

A purposive sampling approach by establishing clear inclusion criteria was used [25, 26], to ensure the selection of participants who could contribute rich and relevant data. Recruitment of participants was guided by pragmatic considerations, particularly the feasibility of conducting in-person interviews. The invitation to participate was sent out by e-mail distributed through the chief dental officers of PDS in counties in Eastern Norway. Dental hygienists and dentists working in PDS were invited to participate in the study. Inclusion criteria consisted of at least 1 year of experience from PDS and a high level of proficiency in spoken Norwegian. A total of eight (8) dental professionals were included, and interviews were conducted using a recording device. Background characteristics are presented in Figure 1. Half of the participants were clinic managers, and most participants worked in district areas, as opposed to cities. All participants were female, with a median age of 44 years (24–65).

### Interviews, transcription, and ethics

An interview guide was developed based on a literature review, the first author’s professional experience from the field and feedback from the research team. The guide is structured around five main themes – interprofessional collaboration, communication, dental services, resource allocation, and preventive efforts. Open-ended questions were used to encourage participants to provide detailed accounts and concrete examples from their professional practice. This approach aimed to elicit in-depth insights and uncover new dimensions of interprofessional collaboration that might not have been anticipated in advance [25]. The interview guide functioned as a flexible framework rather than a rigid script, allowing for the exploration of unfamiliar topics during the interviews. After the first two interviews, the guide was adjusted according to new themes that became apparent.

The participants were informed about the aims and purpose of the project both in writing and orally. Before each interview, the participants were given the opportunity to read the information sheet again and ask questions if there were any. Written consent was obtained from participants before the interviews, which were conducted by the first author, who had prior experience in qualitative research. Interview facilities were chosen by the participants, either at their own clinic (*n* = 6) or at the principal investigator’s workplace (*n* = 2). After each interview, a field note was made. Following each interview, records were transcribed verbatim and deidentified, and both were stored encrypted on USB drive owned by the Oral Health Centre of Expertise in Eastern Norway.

The study was carried out in compliance with the ethical principles set forth in the Declaration of Helsinki and was approved by the Norwegian Agency for Shared Services in Education and Research (No. 98121). In addition, ethical approval was given from Master Review Board at the University of Oslo. Participants were reminded that their professional duty of confidentiality, as mandated for healthcare personnel, remained applicable throughout the interview. The Consolidated Criteria for Reporting Qualitative Research (COREQ) were followed to ensure methodological rigor throughout the study [28], and is available in the Supplementary Material (Appendix 1).

### Data analysis

The data acquired from interviews were analyzed using thematic analysis. The analysis was primarily focused on the explicit meanings expressed in the data, while interpretative elements were applied when discussing the findings in relation to the theoretical framework. The first author conducted an inductive thematic analysis following the six-step process described by Braun and Clarke, identifying, analyzing, and reporting patterns in the data [29–31]. The transcripts were coded manually by the first author. Each transcript was organized in a two-column format, with the original text in one column and the corresponding codes in the adjacent column. A second member of the research team reviewed the coding and provided feedback. Coding was then refined. After coding, the codes were organized into preliminary themes. These were discussed and refined within the research team, and final themes were established based on the study’s aims before producing the report. The circular process of the six phases in Braun and Clarke’s thematic analysis [32] facilitated the identification and highlighting of key findings from the interviews. Engaging continuously with the entirety of the data corpus remained an integral aspect throughout all phases of the analysis. This approach ensured a coherent link between the interview material and the presentation of the findings. An awareness was maintained of the potential impact of our preconceptions and reflexivity, as researchers, on both the analytical process and the interpretation of the data. At the beginning of each interview, participants were informed that all aspects of the collaboration were relevant to the study. Throughout the interview process, clarifying questions were systematically employed to probe participants’ accounts and to ensure an accurate understanding of their perspectives. These strategies contributed to enhancing the rigor of the analytical process and supported the trustworthiness of the study’s findings [32].

In the result part, all direct quotations from participants were numbered from P1 to P8, in addition to removing names of municipalities, clinics, or other subjects. All quotations were translated from Norwegian into English using a digital translation tool, followed by careful review and refinement by the research team to ensure linguistic accuracy and preservation of meaning.

## Results

**Figure 2:**
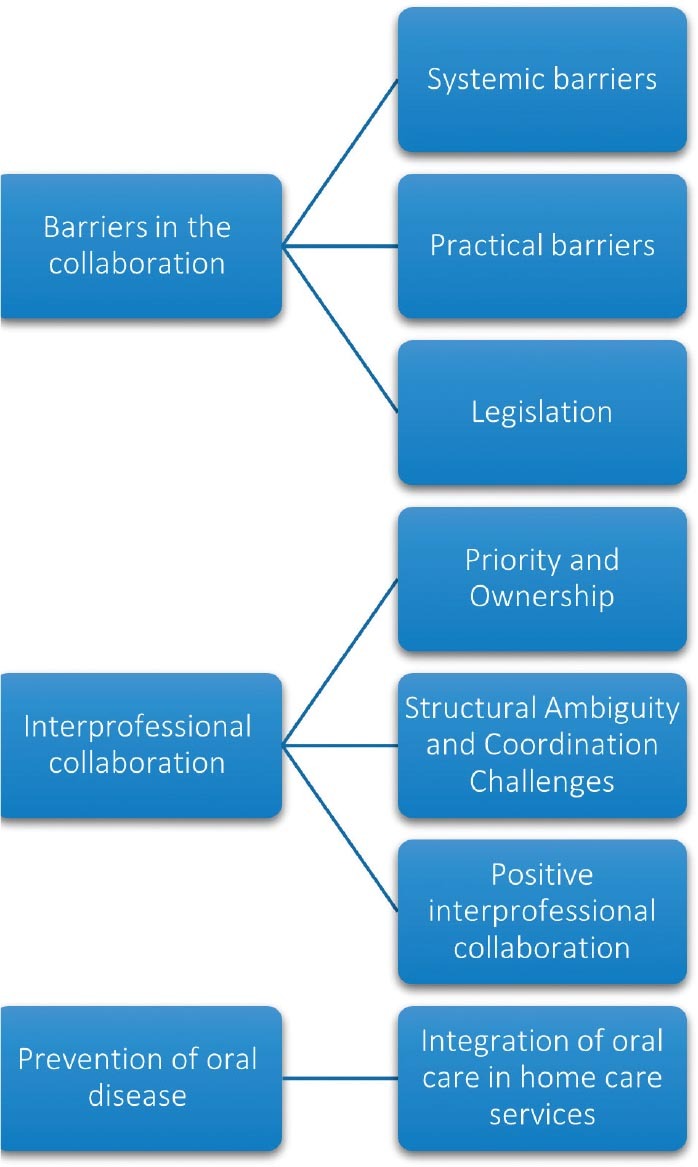
Three main themes are presented with subthemes.

Three main themes were identified through the analysis: Systemic and Practical Barriers in the collaboration, challenges in interprofessional collaboration, and prevention of oral disease, illustrated by connecting subthemes in Figure 2.

### Systemic and practical barriers

All participants described various multilevel challenges in the collaboration between PDS and HCS, including systemic barriers, practice-related obstacles, outdated legislation, and challenges in the interprofessional collaboration. Furthermore, the participants expressed how challenges affected their efforts to prevent oral diseases.

#### Systemic barriers

All participants referred to the formal collaboration agreement underlying the partnership between PDS and HCS, established across multiple levels of the health services administration. Although this formal agreement was intended to clarify responsibilities and streamline collaboration between the two health services, several participants expressed that it had not fulfilled its intended purpose in practice. They identified persistent challenges in establishing a functional collaboration despite the formal agreement and attributed some of these difficulties to factors such as management structures. P5 expressed their experience from the collaboration clearly:

‘Municipal home care services are the services we collaborate with the least effectively.’ (P5)

By describing the collaboration with HCS as particularly problematic, the participant emphasized the severity of these collaborative issues, noting that collaborating with other public services was comparatively easier, in spite of several of them being organized under the same government level as HCS.

One participant explained how PDS was in the process of formalizing the collaboration agreement and had explicitly requested increased involvement from higher management levels in collaboration meetings. This experience mirrored that of another participant, who noted that their collaboration agreement was signed by the senior health manager in HCS as well as the clinic manager at the affiliated dental clinic. The participant emphasized that this process served to legitimize the agreement and facilitate further collaborative efforts.

#### Practical barriers

Several participants identified the lack of a shared digital system as a major challenge, which exacerbated practice-related barriers, as P1 described:

‘We should have a shared platform or some kind of electronic system. Right now, the dental service is completely excluded from that. But there are many barriers that need to be addressed before it becomes a viable option. […] To obtain the necessary information, there are certain details that would be useful to have in advance, before the patients arrive.’ (P1)

P1 highlighted how PDS remained isolated from a shared digital infrastructure with other public health services, such as HCS. This lack of integration across different government levels hindered effective collaboration, particularly concerning digital infrastructure. In the absence of such shared infrastructure, less efficient channels had to be used for secure information sharing. This situation adversely affected both the exchange of information and the continuity of oral health care. Some participants further explained the current methods of communication, which included the use of postal services or telephone, additionally complicating the collaboration. They also emphasized the outdated nature of these methods, particularly since certain situations in the dental clinic require specific information to ensure the provision of appropriate dental treatment.

These findings expressed the need for improved integration between HCS and PDS to facilitate both secure and effective communication and information sharing, thereby strengthening the interagency collaboration. P5 elaborated on this point:

‘Ideally, they should have sent a form, or when updating their lists and approving dental services, the information should have been automatically integrated into our system. That’s how it should be. In a way, these lists should communicate with each other.’ (P5)

As P5 pointed out, it was seen as crucial to establish a system to notify PDS when the users of HCS accepted dental services, enabling PDS to schedule appointments. Both P1 and P5 recommended implementing a shared digital platform to enable rapid and secure information exchange between PDS and HCS, believing that such a digital platform would substantially improve interagency communication.

#### Dental Health Services Act – in need of updates

In addition to systemic challenges in the collaboration between PDS and HCS, participants also encountered legal barriers arising from the Dental Health Services Act (DHSA) of 1984. Under this act, municipal home care recipients are statutorily entitled to oral health care in PDS. Several participants pointed out that the composition of HCS users had changed compared to when the DHSA was enacted, with a broader age range, and an increased proportion of individuals with mental health conditions. P4 described the composition of users as follows:

‘There is a scale, ranging from having perfectly healthy teeth to facing significant challenges for them to come here to the clinic.’ (P4)

P4’s account underscored the heterogeneity of the user group, including younger individuals who retained their own teeth longer than previously. Consequently, these users require more preventive oral health care rather than predominantly treatment-oriented services. P5 further observed that the mandatory 3-month waiting period appeared superfluous, given that most users were anticipated to require lifelong HCS support. Moreover, the participant highlighted that ambiguous wording in the legislation made it difficult for both dental professionals and home care professionals to identify eligible users, adding further complexity to the collaboration.

As a result, several of the participants expressed a clear need for legislative updates that would more accurately reflect current societal conditions, both in terms of the timeframe for granting users their rights, and regarding who should be entitled to them.

### Challenges in interprofessional collaboration

Beyond the systemic and practical challenges, participants highlighted specific issues concerning interprofessional collaboration.

#### Priority and ownership

Different levels of authority in HCS and a lack of clear accountability and responsibility within the formal framework were pointed out as a challenge by some of the participants. They described how this structural deficiency impacted collaboration, particularly when attempting to schedule meetings with HCS. For instance, P3 expressed trying to arrange a meeting with the HCS over a 6-month period, only to have all efforts decline. This created an impression that collaboration meetings were not a priority for the HCS, as P3 explained:

‘I believe they deprioritize it. The collaboration meeting was supposed to be attended by the managers, but they didn’t show up. […] It has been evident that dental health is not a priority for them. They don’t see it as an important part of their daily work.“’ (P3)

The failure to schedule regular collaboration meetings weakened the interprofessional relationship between the two health services according to the participant.

#### Structural ambiguity and coordination challenges

Even when meetings were arranged, the absence of appropriate management representatives, despite explicit requests, undermined collaborative efforts. The inability to clarify roles and responsibilities in the collaboration between the PDS and HCS contributed to fragmented and less effective coordination and management of dental services for HCS users.

Furthermore, P5 described a particular experience that gave the impression that HCS did not consider oral care a vital aspect of their responsibilities in home care nursing:

‘The users do not interact with home-based services; we complete feedback forms after their visits with us. Occasionally, we send these forms to home-based services. […] Not long ago, we received a call from the home nursing service asking, “What on earth are we supposed to do with this form?“’ (P5)

An insufficient understanding of the relevance of oral health feedback forms from the PDS to HCS reflected the ineffective information flow across different management levels. In addition, it indicated how oral care among users was not a priority among health personnel in HCS.

‘Our collaboration agreement states that the home nurse is responsible for this. However, how they implement it and whether the primary contact ensures that patients receive the offer, I don’t know – that is up to them. But they have mentioned a drawback: the offer is not provided until the patient has been in home care for three months. By that point, important details may easily be forgotten. Additionally, there is no alert in their records reminding them, “It’s been three months, you need to ask about dental services.“’ (P8)

As P8 noted, the mandatory 3-month waiting period often delays timely access to essential oral care for the users of HCS. Further, P8 explained that such delays, or, in some cases, complete omission of dental services, occurred because users did not receive the necessary information from HCS once the waiting period had elapsed.

The absence of a standardized routine or system for identifying and following up with eligible users after the 3-month period posed a challenge for HCS to fulfill their responsibilities of informing users about free dental health services. Moreover, the unclear division of responsibilities between the two health services further compounded these delays. According to several participants, this ambiguity in responsibility was one of the key factors contributing to the failure of dental services to reach all users of HCS effectively.

#### Positive interprofessional collaboration

Despite several challenges in the collaboration, two participants also found positive outcomes, particularly where consistent staffing and regular interagency meetings were in place.

‘It is important to engage with people and maintain continuity – to recognize faces. The connection becomes stronger when we have met them, and they have met us, allowing them to see who we are, how we think, and how we work. I believe this fosters positive ripple effects.’ (P4)

Such an approach, P4 noted, builds rapport and understanding, which are crucial for effective interprofessional collaboration across different service levels. Regular meetings and consistency in staff facilitated the collaboration and strengthened the interprofessional relationship positively. P8 shared similar experiences, emphasizing that familiarity and continuity – achieved by working in the same public dental clinic for several years – helped establish stronger relationships and trust between personnel in the two health services. P4 further described that the collaboration with HCS management had improved over time, noting that anchoring it at a higher management level signaled a prioritization of this initiative.

### Prevention of oral disease

Regarding the prevention of oral diseases among HCS users, most participants identified various challenges. P5 highlighted the seriousness of municipal home care’s insufficient attention to users’ oral health:

‘They have become too frail to seek the help they need. It is deeply concerning that this issue has been neglected for so many years. What makes it even more serious is that the responsibility rests with the municipality, which is accountable for the well-being of the entire user, including their dental health. Yet, they have failed to take on that responsibility, leaving it to the dental health services instead. Of course, our role is preventive care, but we only see patients when they require treatment at the clinic. It’s not just me who recognizes this; everyone working in dental health services across Norway sees it. Now, more politicians and healthcare professionals are also starting to acknowledge the problem.’ (P5)

P5’s perspective illustrates how users typically seek dental services only when dental treatment is required, rather than engaging in preventive measures. Moreover, P5 contended that HCS should take on a more comprehensive role by integrating oral health into its scope of care. Two other participants shared similar experiences, indicating that essential oral health information and recommendations did not consistently reach HCS – partly because users visited the clinic independently, and partly because the information did not reach the appropriate HCS personnel. This finding highlights the need for HCS to take on greater responsibility for users’ oral health, thereby becoming a more active stakeholder in collaboration with the PDS.

Participants also noted obstacles in accessing oral care, as many users struggled to visit clinics independently. Consequently, efforts were directed toward preventing oral diseases outside the clinic setting, as P8 pointed out:

‘We must continue to dedicate significant time outside the clinic. Oral health is not created in the clinic – it is shaped where people live and go about their daily lives.’ (P8)

Prevention was thus portrayed as taking place across multiple societal levels and contexts, primarily through daily oral hygiene routines, dietary practices, and professional guidance provided by HCS and dental professionals.

#### Integrating oral care into HCSs

Despite various challenges, half of the participants also acknowledged certain positive aspects. Two participants described conducting home visits in collaboration with HCS personnel. Although home visits limited the scope of dental treatment, they facilitated oral disease prevention by allowing dental professionals to assess users’ oral health and support home care staff in integrating oral health into daily care plans. Consequently, these visits were valued for their potential to enhance collaboration with health personnel in HCS and improve users’ oral health management. However, although beneficial, such initiatives were resource-intensive and lacked broader organizational support for wider implementation at the time of the study. Indeed, only two participants took part in these home visits.

## Discussion

### Structural and organizational barriers to collaboration

This study explored dental professionals’ experiences collaborating with HCS, identifying the organizational divide between HCS and PDS as a key barrier. Although integrated care has been a national priority since the Coordination Reform [13], participants indicated that collaboration challenges persisted. In Norway, the two services operate as structurally distinct systems with different responsibilities and target groups. According to Luhmann’s systems theory, such divisions can function as operationally closed systems, thus shaping how services interpret and respond to information [33]. Furthermore, divisions related to professional roles, expertise, and organization influence how the services interact with one another and where responsibility is executed. Despite a formal collaboration agreement, several participants noted persistent barriers. One highlighted that revising the agreement could strengthen collaboration and clarify stakeholder accountability.

On a more practical level, the lack of a shared digital platform was described as a major barrier to the collaboration on both systemic and practical levels. Similar challenges in cross-sectoral coordination of care for dependent patients have been reported internationally [34]. This was linked to separate administrative systems, each governed by different political bodies [11, 12]. These findings illustrate how structural and administrative fragmentation poses as obstacles to the integration of health services, such as PDS and HCS, both nationally and internationally. Moreover, there is a need to enhance collaborative integrated care. Recognizing these fundamental differences can be essential in the future management of health services. Valentijn et al. [35] propose a framework that delineates integration across micro, meso, and macro levels within health systems. The findings of this study reveal challenges at both macro and micro levels in the collaboration between PDS and HCS. While some participants expressed effective collaboration with HCS leadership, others experienced limited engagement, particularly at the operational level. These discrepancies underscore the need for a structured approach to intersectoral integration. Applying Valentijn’s framework may support the development of more cohesive and equitable collaboration across health services at a national level.

### Legislation

A key finding from this study was that participants perceived current legislation as misaligned with contemporary needs. Eligibility for PDS requires receiving municipal HCS for at least 3 months [9, 12, 36], yet oral health issues often arise earlier – when users are still living at home [2, 5]. This transitional phase is managed inconsistently across regions in Norway, resulting in unequal access to dental services [37], and only about one in five eligible home care users utilize PDS [8]. Further research indicates that users of HCS have substantial needs for emergency dental treatment, which suggests that preventive oral health care is being deprioritized during appointments at PDS [22]. Since prevention is most effective when initiated early, several participants argued that users likely to receive long-term home care should gain access to PDS from the moment such a decision is made. To address this argument, the Dental Health Act must reflect the contemporary needs of society regarding the heterogeneity of users of HCS, and more resources should be allocated towards the prevention of oral diseases.

### Responsibilities

The specialization of health services allows for efficient task management, but complex needs among HCS users often require interdisciplinary care – an issue evident in Norway’s functionally differentiated healthcare system [38]. Research shows that older adults receiving home care frequently suffer from oral health issues impacting quality of life [39], underscoring the need for better collaboration between PDS and HCS. Although HCS has taken on broader coordination roles due to decentralization and an aging population [40], our study found that oral health was not consistently prioritized, even when flagged by dental professionals.

This aligns with findings of role uncertainty among care personnel regarding their responsibilities in this area [41]. Despite overlapping scopes of practice, no professional boundary conflict was observed in this current study from the dental professionals’ perspective, suggesting that HCS staff recognize the expertise of dental professionals and do not seek to claim ownership over oral health tasks [42, 43]. Instead, the lack of clarity appears to stem from systemic ambiguity in role definitions. Furthermore, a previous report has described how leaders of the HCS experienced functioning collaboration with PDS, while at the same time, leaders of PDS experienced challenges [17]. This discrepancy in perceptions of the collaboration between HCS and PDS leadership underscores the systemic ambiguity in relation to responsibilities, further complicating effective collaboration in oral health care for users of HCS. In addition, participants highlighted how meetings were cancelled or poorly attended, limiting opportunities for relationship-building [44]. High staff turnover in home care, especially in larger municipalities, further challenged continuity and required repeated efforts to inform new staff about PDS. Some participants emphasized that personal attributes – such as initiative and persistence – were crucial in maintaining collaboration, supporting previous research highlighting the role of openness and engagement in interprofessional teamwork [45]. When each health service operates based on different assumptions regarding responsibilities and expectations, it poses a significant barrier to the integration of health services [46]. Clearer guidelines and role definitions could enhance interprofessional collaboration and improve the prevention of oral diseases, as suggested in previous research [19].

### Prevention

Both the dental service and municipal home nursing care are legally mandated to prioritize prevention [11, 12, 14]. Several participants criticized the 3-month eligibility delay for dental services among older adults with expected long-term care needs, noting it undermines early prevention. Integrating oral health into routine home care was perceived as essential, a view supported by research highlighting the life-course importance of oral health [47]. A recent Norwegian study found that limited training among HCS personnel hampers preventive efforts, while education and formal instruction improve confidence and outcomes [48]. Strengthening oral health-related education among nurses and other home care personnel may therefore be an important step toward improving preventive oral care for older adults receiving HCS. Emphasizing stronger interprofessional connections, focusing on the exchange of knowledge could strengthen the collaboration and benefit the users of HCS.

Strong interprofessional relationships are essential for effective collaboration [49], and two participants expressed positive experiences with home visits alongside HCS personnel. Although resource-intensive, these visits proved effective in fostering collaboration, mutual understanding, and user-centered preventive efforts. Despite structural constraints, they were seen as important and valuable for frail older adults, who are particularly vulnerable to oral disease [6], and where early intervention may improve health and quality of life [50]. This aligns with recent recommendations to strengthen preventive and interprofessional approaches in oral care for older adults, particularly those reliant on HCS [51].

### Strengths and limitations

All participants in this study were women. This imbalance may limit variations of perspectives presented in the study and potentially affect the transferability of the findings. Additionally, the small sample size may further constrain generalizability. However, as the aim of qualitative research is to provide in-depth insight into experiences and perspectives, rather than to achieve statistical representativeness, the sample size was considered appropriate for the exploratory purpose of this study. The interview guide was not pilot tested prior to data collection, which may have limited its clarity and methodological rigor. The interviews yielded rich, detailed, and nuanced narratives that contributed meaningfully to the study’s analytical depth.

Rigor was enhanced through several methodological decisions. Braun and Clarke’s reflexive thematic analysis was conducted iteratively, moving between transcripts, codes, and themes. This process helped challenge interpretations and refine the analytical process. The research team reviewed the themes and contributed to analytic discussions, supporting reflexivity and reducing the risk of interpretations being shaped solely by the first author’s professional background. The first author’s professional experience may have influenced the interpretation of the data, and such pre-understandings may enrich and shape qualitative analysis.

Notably, participants’ own critical reflections on their role in the collaboration were not deeply explored in this study. The findings may therefore reflect a tendency to attribute challenges to actors or structures outside the participants’ own service, consistent with Luhmann’s systems theory. At the same time, the contrasts in participants’ accounts strengthened the findings by illustrating both positive and negative aspects of the collaboration, as well as reflecting the varied motivations for participation.

## Conclusions and implications

This study shows that structural, administrative, and legislative barriers continue to impede collaboration between the PDS and HCS in Norway. Despite national strategies promoting integrated care, fragmentation and role ambiguity remain. The findings suggest that organizational boundaries influence how responsibilities are interpreted, limiting coordination – particularly in preventive oral care. Although interprofessional conflict was not reported, unclear mandates and weak communication structures led to inconsistent engagement. Participants also criticized outdated legislation that delays access to PDS for frail older adults, undermining timely prevention and failing to reflect current needs. Strengthening collaboration in a changing healthcare landscape requires both infrastructural support and alignment between organizational frameworks and contemporary oral health demands. Interprofessional education and shared digital systems may enhance continuity and trust. Additionally, there is a need for research presenting home care professionals’ perspectives on the collaboration. Positive examples, such as joint home visits, demonstrate the potential for user-centered care, although these practices are possibly resource-intensive and not systematized. There is an urgent need for increased knowledge and research to guide policy and organizational development. Addressing these barriers is essential to secure preventive oral health care for HCS users moving forward.

## Author contributions

The study was designed by IVK under the supervision of MMUS and LF, who also contributed to the development of the interview guide. IVK conducted the interviews and carried out the analysis with guidance from LF and MMUS. The first draft of the article was written by IVK and then revised by MMUS and LF. All authors approved the final submitted version. IVK also completed the COREQ checklist.

## Supplementary Material





## Data Availability

The data supporting the findings of this study are available from the corresponding author (IVK) upon reasonable request.

## References

[1] WHO. Ageing and health [Internet]. World Health Organization; 2024. [cited 2025 January 25]. Available from: https://www.who.int/news-room/fact-sheets/detail/ageing-and-health

[2] McKenna G, Tsakos G, Burke F, Brocklehurst P. Managing an ageing population: challenging oral epidemiology. Prim Dent J. 2020;9: 14–17. 10.1177/2050168420943063 32940594

[3] Rødseth SC, Høvik H, Schuller AA, Bjertness E, Skudutyte-Rysstad R. Dental caries in a Norwegian adult population, the HUNT4 oral health study; prevalence, distribution and 45-year trends. Acta Odontol Scand. 2023;81:202–10. 10.1080/00016357.2022.2117735 36150007

[4] Almasvandi Y, Ziaei N, Kazeminia M, Abbasi P. Global prevalence of dental caries in the older people, 1991 to 2024: a systematic review and meta-analysis. Saudi Dent J. 2025;19(7–9):31. 10.1007/s44445-025-00039-6 PMC12276169 40682726

[5] Czwikla J, Herzberg A, Kapp S, Kloep S, Schmidt A, Rothgang H, et al. Home care recipients have poorer oral health than nursing home residents: results from two German studies. J Dent. 2021;107:103607. 10.1016/j.jdent.2021.103607 33607197

[6] Henni SH, Skudutyte-Rysstad R, Ansteinsson V, Hellesø R, Hovden EAS. Oral health and oral health-related quality of life among older adults receiving home health care services: a scoping review. Gerodontology. 2023;40:161–71. 10.1111/ger.12649 35943193

[7] Norway. Dental Health Services Act [Tannhelsetjenesteloven]. Oslo: Ministry of Health and Care Services; 1984.

[8] Statistics Norway (Statistisk Sentralbyrå). Tannhelsetenesta 11961: Pasientbehandling i tannhelsetjenesten, etter pasientgruppe, statistikkvariabel, år og region. Oslo: Statistisk Norway; 2024.

[9] Norwegian Government. NOU 2024:18 A Universal dental Service. Oslo: Ministry of Health and Care Services; 2024. [cited 2025 August 5]. Available from: https://www.regjeringen.no/no/dokumenter/nou-2024-18/id3054538/

[10] López R, Smith PC, Göstemeyer G, Schwendicke F. Ageing, dental caries and periodontal diseases. J Clin Periodontol. 2017;44(Suppl. 18):S145–52. 10.1111/jcpe.12683 28266118

[11] Norwegian Government. Act relating to municipal health and care services, etc. [Lov om kommunal helse- og omsorgstjenester mm]. Oslo: Ministry of Health and Care Services; 2012

[12] Norwegian Government. Act relating to dental health services. [Tannhelsetjenesteloven]. Oslo: Ministry of Health and Care Services; 1984.

[13] Ministry of Health and Care Services. Rep. No. 47 to the storting: the coordination reform, proper treatment at the right place and right time. Oslo: Ministry of Health and Care Services; 2009.

[14] Government of Norway. Act relating to public health work (Public Health Act). [Lov om folkehelsearbeid]. Oslo: Ministry of Health and Care Services; 2011.

[15] Helsedirektoratet. Tannhelsetjenesten bør samarbeide med kommuner, helseforetak og statlige virksomheter [Internet]. Oslo: Helsedirektoratet; 2022 [cited 2026 Feb 19]. Available from: https://www.helsedirektoratet.no/retningslinjer/tannhelsetjenester-til-barn-og-unge-020-ar/tverrfaglig-samarbeid-og-brukermedvirkning-om-barn-og-unge-0-20-ar/Tannhelsetjenesten-b%C3%B8r-samarbeide-med-kommuner-helseforetak-og-statlige%20virksomheter?

[16] Seim AS, Kveen E, Jacobsen HN, Olsen RH, Willumsen T. Tannhelse og pasienter med hjemmetjenester. Nor Tannlegeforen Tid. 2014;124:712–17. 10.56373/2014-9-10

[17] Hovden ES, Krona ER, Disch PG. Tannhelsetilbudet til brukere av hjemmebaserte omsorgstjenester med rettigheter etter tannhelsetjenesteloven i region Sør. Oslo: Senter for omsorgsforskning, sør; 2017.

[18] Schluter PJ, Askew DA, McKelvey VA, Jamieson HA, Lee M. Oral health among older adults with complex needs living in the community and in aged residential care facilities within New Zealand. J Am Med Direct Assoc. 2021;22:1177–83.e1. 10.1016/j.jamda.2020.06.041 32736993

[19] Mitchell G, Stark P, Wilson CB, Tsakos G, Brocklehurst P, Lappin C, et al. ‘Whose role is it anyway?’ Experiences of community nurses in the delivery and support of oral health care for older people living at home: a grounded theory study. BMC Nurs. 2023;22:359. 10.1186/s12912-023-01533-0 37798687 PMC10557176

[20] Helse- og omsorgsdepartementet. Leve hele livet - en kvalitetsreform for eldre. [Living your whoe life - a quality reform for older adults]. Oslo; Helse- og omsorgsdepartementet; 2018.

[21] Helse- og Omsorgsdepartementet. tiljgengelighet, kompetanse og sosial utjevning - Framtidas tannhelsetjenester [Accessibility, competence and social equity - the dental health services of the future]. Oslo; Helse- og omsorgsdepartemente; 2007.

[22] Uhlen-Strand MM, Hovden EAS, Schwendicke F, Ansteinsson VE, Mdala I, Skudutyte-Rysstad R. Dental care for older adults in home health care services – practices, perceived knowledge and challenges among Norwegian dentists and dental hygienists. BMC Oral Health. 2023;23:222. 10.1186/s12903-023-02951-x 37069568 PMC10111733

[23] Luhmann N. Sociale systemer: grundrids til en almen teori. København: Hans Reitzel; 2000.

[24] Luhmann N. Sosiologisk teori. Oslo: Akademika; 2013.

[25] Green J, Thorogood N. Qualitative methods for health research. 4th ed. Thousand Oaks, CA: Sage Publications; 2018.

[26] Malterud K. Kvalitative metoder i medisinsk forskning – en innføring. 3rd ed. Oslo: Universitetsforlaget; 2013.

[27] Tjora A. Kvalitative forskningsmetoder i praksis. Oslo: Gyldendal; 2021.

[28] Tong A, Sainsbury P, Craig J. Consolidated criteria for reporting qualitative research (COREQ): a 32-item checklist for interviews and focus groups. Int J Qual Health Care. 2007;19:349–57. 10.1093/intqhc/mzm042 17872937

[29] Braun V, Clarke V. Using thematic analysis in psychology. Qual Res Psychol. 2006;3:77–101. 10.1191/1478088706qp063oa

[30] Braun V, Clarke V. Reflecting on reflexive thematic analysis. Qual Res Sport Exerc Health. 2019;11:589–97. 10.1080/2159676X.2019.1628806

[31] Nowell LS, Norris JM, White DE, Moules NJ. Thematic analysis: striving to meet the trustworthiness criteria. Int J Qual Methods. 2017;16:1609406917733847. 10.1177/1609406917733847

[32] Braun V, Clarke V. Thematic analysis: a practical guide. London: SAGE Publications; 2022.

[33] Rasmussen J. Luhmann anvendt. København: Unge Pædagoger; 2002.

[34] Göstemeyer G, Baker SR, Schwendicke F. Barriers and facilitators for provision of oral health care in dependent older people: a systematic review. Clin Oral Investig. 2019;23:979–93. 10.1007/s00784-019-02812-4 30707299

[35] Valentijn PP, Schepman SM, Opheij W, Bruijnzeels MA. Understanding integrated care: a comprehensive conceptual framework based on the integrative functions of primary care. Int J Integr Care. 2013;13:e010. 10.5334/ijic.886 23687482 PMC3653278

[36] Norwegian Directorate of Health (Helsedirektoratet). Tannbehandling til eldre, langtidssyke og uføre [Dental care for the elderly, chronically ill, and persons with disabilitie] [Nettdokument]. Norwegian Directorate of Health; 2019 [updated 2024 Sept 18. Nettdokument]. [cited 2024, December 16]. Available from: https://www.helsedirektoratet.no/lov-og-forskrift/tannhelsetjenesteloven/tannbehandling-til-pasienter-i-gruppe-c

[37] Strand GV. Tannløs og lovløs. Nor Tannlegeforen Tid. 2018;7:118. 10.56373/2018-7-13

[38] Vik E, Hjelseth A. Integration of health services – eight theses on coordination in a functionally differentiated healthcare system. Norwegian J Soc Res. 2022;63:122–40. 10.18261/tfs.63.2.3

[39] Hassan HI, Uhlen-Strand MM, Ansteinsson V, Hellesø R, Hovden EAS, Skudutyte-Rysstad R. Self-reported oral health and oral health-related quality of life among older adults receiving home care services in South-eastern Norway. Acta Odontol Scand. 2025;84:165–73. 10.2340/aos.v84.43425 40202116 PMC12020431

[40] Melby L, Obstfelder A, Hellesø R. ‘We tie up the loose ends’: homecare nursing in a changing health care landscape. Glob Qual Nurs Res. 2018;5:2333393618816780. 10.1177/2333393618816780 30574532 PMC6295756

[41] Idris S, Aghanwa S, O’Halloran J, Durey A, Slack-Smith L. Homebound oral care for older adults: a qualitative study of professional carers’ perspectives in Perth, Western Australia. Gerodontology. 2024;41:94–100. 10.1111/ger.12704 37454389

[42] Abbott A. The system of professions: an essay on the division of expert labor. Chicago, IL: University of Chicago Press; 1988.

[43] Schönfelder W. Legenes posisjon i et tverrfaglig landskap: Samhandlingsreformen i et profesjonsteoretisk perspektiv. In: Tjora A, Melby L, editors. Samhandling for helse. Oslo: Gyldendal; 2013. p. 54–78.

[44] Vik E. Coordination between health care professions – a scoping review. Norwegian J Welfare Res. 2018;21:119–47. 10.18261/issn.2464-3076-2018-02-03

[45] San Martín-Rodríguez L, Beaulieu M-D, D’Amour D, Ferrada-Videla M. The determinants of successful collaboration: a review of theoretical and empirical studies. J Interprof Care. 2005;19:132–47. 10.1080/13561820500082677 16096151

[46] Auschra C. Barriers to the integration of care in inter-organisational settings: a literature review. Int J Integr Care. 2018;18:5. 10.5334/ijic.3068 PMC5887071 29632455

[47] Slack-Smith L, Ng T, Macdonald ME, Durey A. Rethinking oral health in aging: ecosocial theory and intersectionality. J Dent Res. 2023;102:844–8. 10.1177/00220345231175061 37314086 PMC10345992

[48] Hassan HI, Ansteinsson VE, Dalbak ET, Skudutyte-Rysstad R, Hellesø R, Mdala I, et al. Oral healthcare beliefs among home care services personnel; a cross-sectional study in south-eastern Norway. BMC Health Serv Res. 2024;24:1090. 10.1186/s12913-024-11534-7 39294684 PMC11409571

[49] Orvik A. Organisatorisk kompetanse: innføring i profesjonskunnskap og klinisk ledelse. 2nd ed. Oslo: Cappelen Damm Akademisk; 2015.

[50] van de Rijt LJ, Stoop CC, Weijenberg RA, de Vries R, Feast AR, Sampson EL, et al. The influence of oral health factors on the quality of life in older people: a systematic review. Gerontologist. 2020;60:e378–94. 10.1093/geront/gnz105 31729525

[51] WHO. Oral health conditions [Internet]. World Health Organization; 2020. [cited 2025, May 9]. Available from: https://www.who.int/news-room/fact-sheets/detail/oral-health

