# Supplementary material for: Exploring dental professionals’ experiences of interprofessional collaboration with home care services
Source: Acta Odontol Scand. 2026 Apr 24;85:45934. doi: 10.2340/aos.v85.45934 (PMC13123240; doi:10.2340/aos.v85.45934)
Supplement: Supplementary file 1 [file AOS-85-45934-s1.pdf]

Supplementary material has been published as submitted. It has not been copyedited or typeset by Acta Odontologica Scandinavica.

Table 1
Consolidated criteria for reporting qualitative studies (COREQ): 32-item checklist

No	Item	Guide questions/description	Response
**Domain 1: Research team and reflexivity**			
Personal Characteristics			
1.	Interviewer/facilitator	Which author/s conducted the interview or focus group?	Ingrid Volden Klepaker conducted the interviews
2.	Credentials	What were the researcher's credentials? *E.g. PhD, MD*	IVK: Msc<br>Marte-Mari Uhlen Strand: PhD<br>Lena Fauske: MA PhD
3.	Occupation	What was their occupation at the time of the study?	IVK: Dental Hygienist, research coordinator<br>MMU: Senior researcher<br>LF: Senior researcher
4.	Gender	Was the researcher male or female?	All three were female
5.	Experience and training	What experience or training did the researcher have?	IVK: Dental Hygienist, some prior experience with semistructured interviewing.<br>MMU: Public Dental Health research, oral health research, research, gerodontology research<br>LF: Qualitative research, experienced counsellors for several master/PhD-students

No	Item	Guide questions/description	Response
Relationship with participants			
6.	Relationship established	Was a relationship established prior to study commencement?	No
7.	Participant knowledge of the interviewer	What did the participants know about the researcher? e.g. *personal goals, reasons for doing the research*	The participants were told about the interviewers' professional background, and the reason for conducting the study
8.	Interviewer characteristics	What characteristics were reported about the interviewer/facilitator? e.g. *Bias, assumptions, reasons and interests in the research topic*	Dental Hygienist<br>Former experience from public dental services<br>Some experience from collaboration with home care services
**Domain 2: study design**			
Theoretical framework			
9.	Methodological orientation and Theory	What methodological orientation was stated to underpin the study? *e.g. grounded theory, discourse analysis, ethnography, phenomenology, content analysis*	An exploratory qualitative design with a reflective, interpretive approach to understanding experiences and thematic analyzes underpinned the study
Participant selection			

No	Item	Guide questions/description	Response
10.	Sampling	How were participants selected? *e.g. purposive, convenience, consecutive, snowball*	Participants were purposive selected by IVK.
11.	Method of approach	How were participants approached? E.*g. face-to-face, telephone, mail, email*	Face to Face
12.	Sample size	How many participants were in the study?	Eight
13.	Non-participation	How many people refused to participate or dropped out? Reasons?	No drop out
Setting			
14.	Setting of data collection	Where was the data collected? e.*g. home, clinic, workplace*	Interviews at clinic: six Interviews at first author's workplace: two
15.	Presence of non-participants	Was anyone else present besides the participants and researchers?	No
16.	Description of sample	What are the important characteristics of the sample? *e.g. demographic data, date*	The study included eight women. The participants median age was 44 years (range: 24–65 years)
Data collection			
17.	Interview guide	Were questions, prompts, guides provided by the authors? Was it pilot tested?	The interview guide was drawn on relevant literature and IVKs previous experiences with working in public dental services,

No	Item	Guide questions/description	Response
			collaborating with home care services. The interview guide was not pilot tested.
18.	Repeat interviews	Were repeat interviews carried out? If yes, how many?	No
19.	Audio/visual recording	Did the research use audio or visual recording to collect the data?	All interviews were audiotaped and transcribed
20.	Field notes	Were field notes made during and/or after the interview or focus group?	Yes, field notes were made after the interviews
21.	Duration	What was the duration of the interviews or focus group?	22–60 minutes
22.	Data saturation	Was data saturation discussed?	No, but the sample selection proved to be adequate in this study, as the narratives were rich and full of nuanced examples.
23.	Transcripts returned	Were transcripts returned to participants for comment and/or correction?	No
**Domain 3: analysis and findings**z			
Data analysis			
24.	Number of data coders	How many data coders coded the data?	The first author did the coding, and a second member

No	Item	Guide questions/description	Response
			of the research team reviewed the coding.
25.	Description of the coding tree	Did authors provide a description of the coding tree?	No
26.	Derivation of themes	Were themes identified in advance or derived from the data?	Themes were derived from the data
27.	Software	What software, if applicable, was used to manage the data?	No software was used
28.	Participant checking	Did participants provide feedback on the findings?	No
Reporting			
29.	Quotations presented	Were participant quotations presented to illustrate the themes / findings? Was each quotation identified? e.*g. participant number*	Participant quotations were included to illustrate the themes, with each participant assigned a unique ID to maintain confidentiality. To further protect anonymity, neither gender nor age was associated with the quotes.
30.	Data and findings consistent	Was there consistency between the data presented and the findings?	While the data showed overall consistency, variations within the material provided rich and nuanced examples that helped illuminate participants' experiences from their own perspectives

No	Item	Guide questions/description	Response
31.	Clarity of major themes	Were major themes clearly presented in the findings?	Yes, three main themes were presented
32.	Clarity of minor themes	Is there a description of diverse cases or discussion of minor themes?	Yes, deviations within the material were presented
