# Supplementary material for: Exploring dental professionals’ experiences of interprofessional collaboration with home care services
Source: Acta Odontol Scand. 2026 Apr 24;85:45934. doi: 10.2340/aos.v85.45934 (PMC13123240; doi:10.2340/aos.v85.45934)
Supplement: Supplementary file 2 [file AOS-85-45934-s2.pdf]

Supplementary material has been published as submitted. It has not been copyedited or typeset by Acta Odontologica Scandinavica.

Intervjuguide

Innledning:

1. Hvordan er det å jobbe med de pasientene som er brukere av kommunal hjemmesykepleie?
2. Hvordan vil du beskrive din erfaring med brukere av kommunal hjemmesykepleie?
3. Hvordan oppfatter du tannhelsen til brukere av kommunal hjemmesykepleie?

Samarbeid:

1. Hvordan vil du beskrive samarbeidet mellom tannklinikken du jobber på og den kommunale hjemmesykepleien?
    - Hvem deltar på samarbeidsmøter med kommunal hjemmesykepleie?
    - Hvor ofte foregår dette?
    - Hvem organiserer/tar initiativ til disse møtene?
2. Hvordan opplever du samarbeidsmøtene?
3. Hvordan evalueres samarbeidsavtalene eller samarbeidet?
4. Hvordan vurderer du samarbeidet mellom tannklinikken og kommunal hjemmesykepleie?
    - Hva fungerer bra i samarbeidet?
    - Hva fungerer mindre bra i samarbeidet?
5. Dersom undervisning til ansatte i kommunal hjemmesykepleie: Hvordan opplever du dette?

Kommunikasjon

1. Hvordan syns du kommunikasjon mellom tannklinikken og den kommunale hjemmesykepleien fungerer?
2. Støttespørsmål:
    - Informasjon om tannhelsetilbudet?
    - Timeavtaler
    - Følge av pasienter
    - Medisinlister?

3. I hvilke andre sammenhenger møter du ansatte fra den kommunale hjemmesykepleien?

Tilbudet

1. Hvordan får brukere av kommunal hjemmesykepleie får informasjon om sine rettigheter i Den Offentlige Tannhelsetjenesten.
2. Hvordan får dere på tannklinikken beskjed fra kommunal hjemmesykepleie om nye pasienter som har rett på tilbudet under brukere av kommunal hjemmesykepleie?
    - Hvordan får dere på tannklinikken beskjed om pasienter som ikke lenger har rettigheter?
3. Ansvar: Hvem har ansvar for at tannhelsetilbudet fra Den Offentlige Tannhelsetjenesten når frem til brukerne av kommunal hjemmesykepleie?
    - Hvilke parter er involvert i å informere om tilbudet til brukerne av kommunal hjemmesykepleie?
    - Hvilket ansvar har kommunal hjemmesykepleie i forhold til brukerne?
    - Hvordan kan man tydeliggjøre ansvaret på en bedre måte?

Ressurser

1. Hvordan syns du ressursene på tannklinikken/Den Offentlige Tannhelsetjenesten er fordelt med tanke på organisering av tannhelsetilbudet til brukere av kommunal hjemmesykepleie?
    - Hvordan kan man eventuelt organisere dagens ressurser annerledes?

Forebygging:

1. Hvordan jobber du med forebygging av orale sykdommer iblant brukerne av kommunal hjemmesykepleie?
    - Hvilke erfaringer har du med å anbefale kommunal hjemmesykepleie at brukerne bør motta hjelp til tannpleie fra hjemmesykepleien?

Avrunding av intervjuet

1. Er det noe du savner som du synes jeg burde spurt deg om?
2. Kan jeg ta kontakt hvis det dukker opp spørsmål jeg har glemt å stille eller ønsker å få utdypet?
3. Hva var det som gjorde at du hadde lyst til å bidra i dette intervju?

4. Hvordan opplevde du denne intervjusituasjonen?
    - Hva var bra med den?
    - Hva var mindre bra?
    - Hvordan kjente du deg påvirket av meg som intervjuer i intervjusituasjonen? (positivt, negativt)
